# Successful treatment of rituximab-refractory pemphigus vulgaris with Obinutuzumab^؆^

**DOI:** 10.1016/j.abd.2026.501457

**Published:** 2026-08-28

**Authors:** Muhammet Talha Saraç, Merve Aygün Alizada, Handan Merve Erol Mart, Ayşe Boyvat, Hatice Şanlı, Ayşe Öktem

**Affiliations:** Department of Dermatology, Faculty of Medicine, Ankara University, Ankara, Turkey

Dear Editor,

Pemphigus Vulgaris (PV) is an autoimmune blistering disease in which Rituximab (RTX) constitutes the standard first-line therapy for moderate-to-severe disease; however, resistance or hypersensitivity may occur. We report a severe case of RTX-refractory PV demonstrating a rapid clinical response to obinutuzumab.

A patient with a 10-year history of PV presented with extensive mucocutaneous erosions. Prior therapies included high-dose systemic corticosteroids, plasmapheresis, cyclophosphamide, mycophenolate sodium, long-term azathioprine, 59 courses of intravenous immunoglobulin, and RTX, which was initially discontinued because of an anaphylactic reaction. RTX was subsequently reintroduced using a desensitization protocol and administered every six months for four years (eight infusions total); however, persistent blistering necessitated additional IVIG courses without clinical benefit. Owing to ongoing disease activity, obinutuzumab was initiated at a dose of 1000 mg on days 1, 8, and 15 with standard premedication.

The Pemphigus Disease Area Index (PDAI) score decreased from 73 (activity: 60; damage: 13) at baseline to 21 (activity: 12; damage: 9) at one month and further to 7 (activity: 3; damage: 4) at six months, indicating sustained clinical improvement. The PDAI activity component evaluates active erosions, blisters, or new erythema involving the skin, scalp, and mucous membranes, whereas the damage component is scored separately and records post-inflammatory changes, such as hyperpigmentation or erythema in resolving lesions.^1^ To facilitate interpretation, previously proposed PDAI severity thresholds have classified pemphigus as mild, moderate, or severe using different cut-off values. Shimizu et al. defined mild disease as 0–8, moderate disease as 9–24, and severe disease as ≥25,^2^ whereas Boulard et al. proposed PDAI cutoff values of 15 and 45 to distinguish moderate, significant, and extensive pemphigus forms.^3^ Accordingly, our patient’s baseline PDAI score of 73 was markedly elevated and subsequently fell to a substantially lower range after obinutuzumab treatment, with the activity score decreasing from 60 to 12 at one month and to 3 at six months. This reduction reflects a clear decrease in active disease burden, while the parallel decrease in the damage score from 13 to 9 and then to 4 indicates progressive resolution of post-inflammatory sequelae rather than ongoing blistering activity (Fig. 1). Indirect immunofluorescence titers declined from 1:640 before treatment to 1:320 at one month and 1:160 at six months. Aside from transient lymphopenia during the second treatment week, no adverse events were observed. Azathioprine was continued at 100 mg/day as maintenance therapy.

**Fig. 1:**
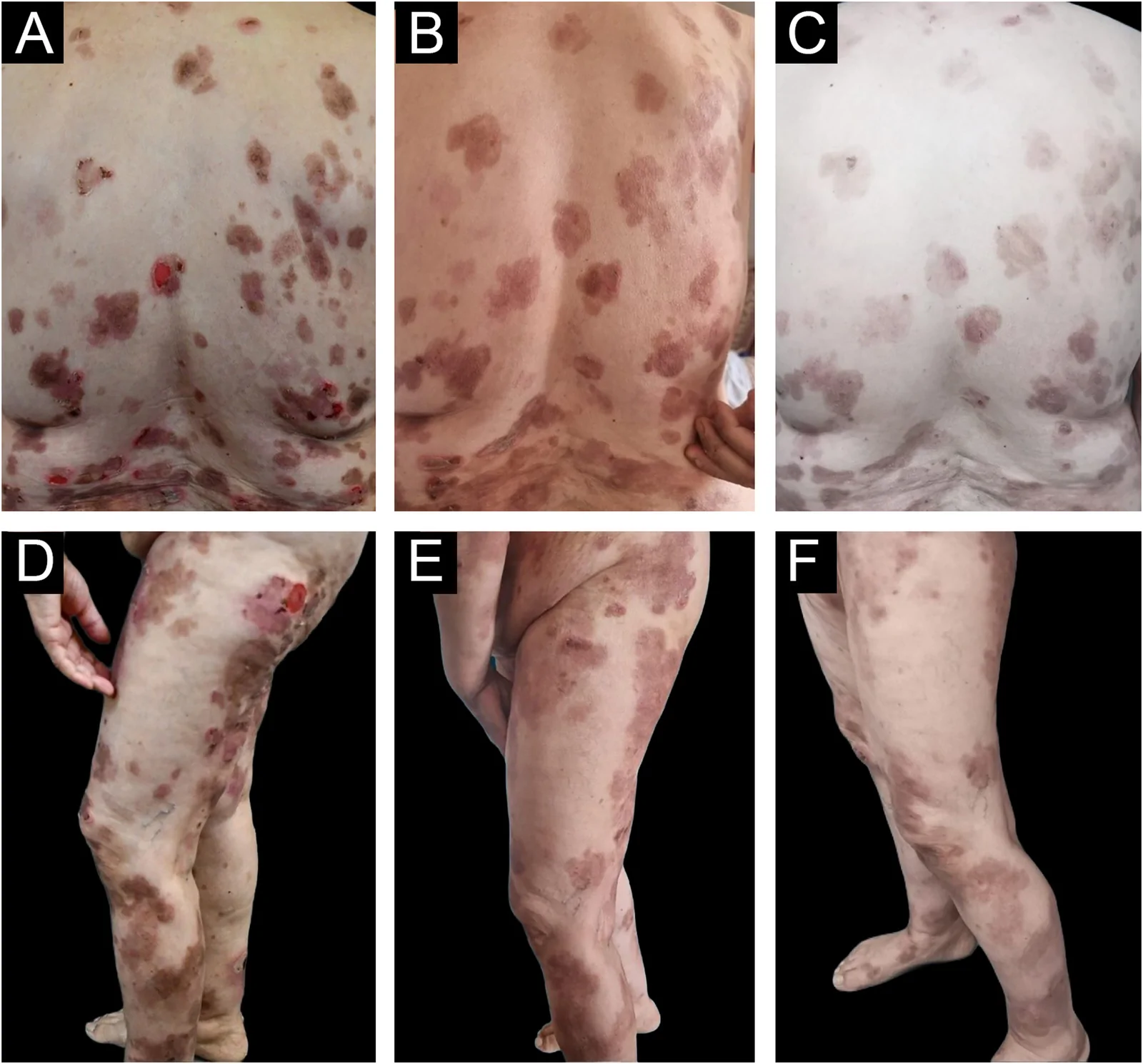
Sequential clinical improvement in pemphigus vulgaris at baseline (A‒D), one month (B‒E), and six months (C‒F). At baseline, multiple erosive plaques involving the trunk and extremities were observed (A‒D), with a PDAI score of 73 (activity: 60, damage: 13), indicating severe disease. After one month of treatment, re-epithelialization of erosions with post-inflammatory hyperpigmentation was evident (B‒E), reflected by a decrease in PDAI to 21 (activity: 12, damage: 9). At six months, further reduction in lesion activity and residual hyperpigmentation demonstrated sustained remission (C‒F), with a PDAI of 7 (activity: 3, damage: 4).

Obinutuzumab is currently indicated for chronic lymphocytic leukemia, follicular lymphoma, and lupus nephritis.^4^ Anti-CD20 Monoclonal Antibodies (MABs) are classified according to their mechanisms of action into type I and type II agents. Type I antibodies (RTX, ofatumumab, ocrelizumab, veltuzumab) mediate B-cell depletion predominantly through complement-dependent cytotoxicity, whereas type II antibodies (obinutuzumab, tositumomab) induce enhanced direct apoptosis and Antibody-Dependent Cellular Cytotoxicity (ADCC) with limited complement activation.^5^, ^6^, ^7^, ^8^ These different mechanisms of action may help overcome RTX-specific resistance mechanisms, including impaired lipid raft localization, incomplete B-cell depletion, and the persistence of autoreactive B-cells within lymphoid compartments.

Obinutuzumab demonstrated clinically meaningful benefit in six cases, including four patients with pemphigus vulgaris who developed rituximab-associated allergic reactions and two patients with lymphoma-associated paraneoplastic pemphigus who showed marked clinical improvement with obinutuzumab-based therapy, despite one subsequent occurrence of bronchiolitis obliterans.^5^, ^6^, ^7^, ^8^

Similarly, in our patient with RTX-refractory PV, obinutuzumab treatment resulted in rapid cessation of blister formation, a clinically meaningful reduction in PDAI scores within weeks, and sustained clinical improvement through six months without the development of infusion-related hypersensitivity reactions. This clinical response may be related to the mechanism of action of obinutuzumab, a humanized type II anti-CD20 MAB engineered with Fc glyco-modifications that enhance FcγRIIIa affinity, leading to markedly augmented ADCC.^5^, ^7^, ^8^ Indeed, rapid and profound B-cell depletion has been demonstrated across both hematologic malignancies and autoimmune diseases.^5^, ^7^

Accumulating evidence from individual case reports suggests that alternative anti-CD20 MABs, including veltuzumab, ocrelizumab, and ofatumumab, confer clinically meaningful efficacy and acceptable safety in RTX-refractory or rituximab-intolerant cases; however, larger prospective studies with extended follow-up are required to confirm these findings and to establish optimal therapeutic strategies.^5^, ^6^, ^7^, ^9^

## ORCID ID

Muhammet Talha Saraç: 0009-0001-7459-2729

Merve Aygün Alizada: 0000-0003-2732-9132

Handan Merve Erol Mart: 0000-0003-3409-8985

Ayşe Boyvat: 0000-0001-7897-8349

Hatice Şanlı: 0000-0002-2960-3126

Ayşe Öktem: 0000-0003-3810-6188

## Financial support

This study received no specific grant from any funding agency in the public, commercial, or not-for-profit sectors.

## Authors’ contributions

Muhammet Talha Saraç: Approval of the final version of the manuscript; critical literature review; data collection, analysis and interpretation; effective participation in research orientation; intellectual participation in propaedeutic and/or therapeutic management of studied cases; manuscript critical review; preparation and writing of the manuscript; study conception and planning.

Merve Aygün Alizada: Approval of the final version of the manuscript; critical literature review; data collection, analysis, and interpretation; effective participation in research orientation; intellectual participation in propaedeutic and/or therapeutic management of studied cases; manuscript critical review; preparation and writing of the manuscript; study conception and planning.

Handan Merve Erol Mart: Approval of the final version of the manuscript, critical literature review, data collection, analysis and interpretation; effective participation in research orientation; intellectual participation in propaedeutic and/or therapeutic management of studied cases; manuscript critical review; preparation and writing of the manuscript; study conception and planning.

Ayşe Boyvat: Approval of the final version of the manuscript; critical literature review; data collection, analysis and interpretation; effective participation in research orientation; intellectual participation in propaedeutic and/or therapeutic management of studied cases; manuscript critical review; preparation and writing of the manuscript; study conception and planning.

Hatice Şanlı: Approval of the final version of the manuscript; critical literature review; data collection, analysis and interpretation; effective participation in research orientation; intellectual participation in propaedeutic and/or therapeutic management of studied cases; manuscript critical review; preparation and writing of the manuscript; study conception and planning.

Ayşe Öktem: Approval of the final version of the manuscript; critical literature review; data collection, analysis and interpretation; effective participation in research orientation; intellectual participation in propaedeutic and/or therapeutic management of studied cases; manuscript critical review; preparation and writing of the manuscript; study conception and planning.

## Research data availability

Does not apply.

## Conflicts of interest

The authors declare that they have no potential conflict of interest regarding the investigation, authorship, and/or publication of this article.
